# The presence of circulating antibody secreting cells and long-lived memory B cell responses to reticulocyte binding protein 1a in *Plasmodium vivax* patients

**DOI:** 10.1186/s12936-021-04015-3

**Published:** 2021-12-20

**Authors:** Piyawan Kochayoo, Pattarawan Sanguansuttikul, Pongsakorn Thawornpan, Kittikorn Wangriatisak, John H. Adams, Francis B. Ntumngia, Patchanee Chootong

**Affiliations:** 1 Department of Clinical Microbiology and Applied Technology, Faculty of Medical Technology, Mahidol University, Bangkok, 10700 Thailand; 2 Center for Global Health and Infectious Diseases Research, University of South Florida, Tampa, FL 33612 USA

**Keywords:** *Plasmodium vivax*, Reticulocyte binding protein 1a, Long-lived memory B cells, Antibody secreting cells

## Abstract

**Background:**

Development of an effective vaccine against blood-stage malaria requires the induction of long-term immune responses. *Plasmodium vivax* Reticulocyte Binding Protein 1a (PvRBP1a) is a blood-stage parasite antigen which is associated with invasion of red blood cells and induces antibody responses. Thus, PvRBP1a is considered as a target for design of a blood-stage vaccine against vivax malaria.

**Methods:**

Both cross-sectional and cohort studies were used to explore the development and persistence of long-lived antibody and memory B cell responses to PvRBP1a in individuals who lived in an area of low malaria endemicity. Antibody titers and frequency of memory B cells specific to PvRBP1a were measured during infection and following recovery for up to 12 months.

**Results:**

IgG antibody responses against PvRBP1a were prevalent during acute vivax malaria, predominantly IgG1 subclass responses. High responders to PvRBP1a had persistent antibody responses for at least 12-month post-infection. Further analysis of high responder found a direct relation between antibody titers and frequency of activated and atypical memory B cells. Furthermore, circulating antibody secreting cells and memory B cells specific to PvRBP1a were generated during infection. The PvRBP1a-specific memory B cells were maintained for up to 3-year post-infection, indicating the ability of PvRBP1a to induce long-term humoral immunity.

**Conclusion:**

The study revealed an ability of PvRBP1a protein to induce the generation and maintenance of antibody and memory B cell responses. Therefore, PvRBP1a could be considered as a vaccine candidate against the blood-stage of *P. vivax*.

**Supplementary Information:**

The online version contains supplementary material available at 10.1186/s12936-021-04015-3.

## Background

Malaria is a serious global public health problem, causing around 229 million cases and an estimated 4,09,000 deaths in 2019 [1]. *Plasmodium vivax* is one of the most prevalent malarial species in the world, found especially in Asia and the Americas [2]. Treatment and control of *P. vivax* have become serious challenges due to drug and vector resistance, wide distribution, antigen variation, relapsing biology and frequent co-infection with *Plasmodium falciparum* [3]. Moreover, naturally acquired immune responses to *P. vivax* are short-lived and biased toward strain-specific immunity [4, 5]. Given these factors, a prophylactic vaccine would add an important tool in strategies to prevent and eliminate malaria.

Blood-stages of the life cycle of *P. vivax* are responsible for the clinical symptoms associated with the infection. Therefore, a vaccine against this stage would reduce parasite load and clinical severity. Several blood-stage antigens that are expressed on merozoites play critical roles during the invasion of red blood cells (RBCs) and are attractive targets for an effective vaccine [6, 7]. One of the leading blood-stage vaccine candidates is *P. vivax* duffy binding protein (PvDBP), a parasite cell surface protein in the erythrocyte binding-like (EBL) invasion protein family [8, 9]. This protein binds to the duffy antigen receptor for chemokines (DARC), a receptor on the surface of the erythrocyte [10]. There are individuals with naturally acquired immunity who possess anti-DBP antibodies that inhibit the DBP-DARC interaction and appear to neutralize *P. vivax* invasion [11]. However, recent studies reported that Duffy-negative individuals are involved in *P. vivax* infection, indicating the existence of an alternative pathway of invasion [12, 13]. Therefore, finding new vaccine candidates with distinct target antigens is necessary.

The *P. vivax* reticulocyte binding protein (PvRBP) family is a group of merozoite proteins that play an important role in parasite invasion of RBCs [14]. It is composed of 11 members, encoded in five full-length genes (*pv*rbp1a, *pv*rbp1b, *pv*rbp2a, and *pv*rbp2b, and *pv*rbp2c), three partial genes (*pv*rbp1p1, *pv*rbp2p1, and *pv*rbp2p2) and three pseudogenes (*pv*rbp2d, *pv*rbp2p1, and *pv*rbp3), based on sequence homology to existing *P. vivax* RBP and *Plasmodium yoelii* Py235 members [15, 16]. Among of them, PvRBP1a is proposed as a blood-stage vaccine candidate as it forms a complex and binds specifically to reticulocytes. However, its cognate receptors remain to be tested further by finding the key receptor-ligand interactions that mediate host cell invasion [17]. Antigenicity of PvRBP1a has been shown in mice as immunization stimulated high-titer antibody responses [18, 19]. In patients, high rates of positivity for anti-PvRBP1a are reported in natural *P. vivax* infection in Papua New Guinea (PNG), Brazil, India and Thailand [19–24]. Previous studies, focusing on IgG antibody profiling, revealed that cytophilic IgG1 and IgG3 are the predominant antibody subclasses in responses to PvRBP1a antigen [21, 24]. These cytophilic antibodies against PvRBP1a may contribute to protection against clinical malaria in a high transmission area of PNG [21]. Moreover, natural human antibodies against PvRBP1a have been shown to inhibit merozoite invasion of reticulocytes [19, 23]. Altogether, PvRBP1a might be a promising candidate as a vaccine against *P. vivax*.

Here, seroprevalence of anti-PvRBP1a antibodies was determined in *P. vivax* patients. Cross-sectional and longitudinal studies were conducted to determine anti-PvRBP1a IgG and IgG subclass levels, and to assess correlation with frequency of memory B cell (MBC) subsets and plasma cells. The development of antibody secreting cells (ASCs) and long-lived MBCs specific to PvRBP1a was evaluated during and after malaria infection. A better understanding of the longevity of humoral immunity against PvRBP1a will be useful in the development of an effective vivax malaria vaccine based on this protein in the future.

## Methods

### Study participants and study design

Cross-sectional and cohort studies were designed to explore the generation of long-term antibody and MBC responses to PvRBP1a in low malaria transmission areas of Southern Thailand (Ranong province). Plasma anti-PvRBP1a antibodies were measured in vivax malaria patients (n  = 68) seen between May 2014 and May 2019. Subjects positive to RBP1a in total IgG (n  = 41) were further analysed for anti-PvRBP1a in IgG subclasses. Of these 41 seropositive patients, 16 patients who were available for monitoring at all time-points in a 12-month cohort study (acute and recovery from infection for 3, 9 and 12 months) were recruited to monitor the longevity of total anti-PvRBP1a IgG responses. The characteristics of participants enrolled in seroprevalence study are shown in Table 1.

**Table 1:** Characteristics of study participants assessed for PvRBP1a antigenicity during acute and recovery phases of vivax malaria

Characteristic	Acute *P.* *vivax* patients					Recovered *P. vivax* subjects				Healthy subjects
	Total IgG				IgG subclasses	Kinetic responses				
	All	HR	LR	NR		3m	9m	12m		
Number	68	11	30	27	41 (of total 68)	16 (of total 68)				43
Age mean ± SD	32.42 ± 14.43	46.56 ± 11.78	31.24 ± 13.25	27.77 ± 13.26	32.42 ± 14.43	47.86 ± 14.14				27.22 ± 6.85
Gender										
Male	45	7	20	18	27	12	12	12		20
Female	23	4	10	9	14	4	4	4		23
Nationality										
Thai	49	8	25	16	33	15	15	15		43
Myanmar	19	3	5	11	8	1	1	1		0
No. of prior infection										
0	68	11	30	27	41	16	15	15		0
1	0	0	0	0	0	0	1	1		0
> 1	0	0	0	0	0	0	0	0		0
No. of recorded re-infections	0	0	0	0	0	0	0	0		0
Parasitaemia (parasite/µL) mean ± SD (range)	5318.81 ± 4213.53 (120–15,000)					0	0		0	0

*HR* high responders, indicates high response to PvRBP1a; *LR* low responders, indicates low response to PvRBP1a; *NR* non-responders, indicates negative response PvRBP1a; *3m, 9m and 12m* times since recovery from *Plasmodium vivax* infection, 3, 9 and 12 months

To explore the relationship of frequency of MBC subsets and plasmablasts/plasma cells with anti-PvRBP1a antibody levels during acute malaria illness, peripheral blood mononuclear cells (PBMCs) from acute patients (n  = 20) were isolated to perform flow cytometric analysis, and plasma was collected for detection of total IgG. Moreover, the presence of PvRBP1a-specific MBCs was investigated in acute patients (n  = 10) and recovered patients (1–3 months, n  = 7; 1–3 years, n  = 7). Healthy volunteers who lived in non-malaria endemic areas (Bangkok, Thailand) were recruited as healthy controls (HC, n  = 55).

The patients who had febrile illness and positive *P. vivax* parasitaemia by microscopic examination of both thick and thin blood smears, and nested PCR were recruited as acute vivax malaria infection. After receiving anti-malarial drug treatment, the patients were enrolled in post-infection phase of *P. vivax* infection. History of previous malaria infections in individual subjects were obtained from the records of the malaria clinic at Vector Borne Disease Unit. Subjects were scheduled for blood sample collection every 3 months for assessment of sub-patent malaria by nested PCR. Staff conducted weekly house to house visits from May 2014 to May 2019 to estimate the incidence of clinical malaria over the study period. Ethical approval was obtained from the Committee on Human Rights Related to Human Experimentation, Mahidol University, Thailand (MUIRB 2012/079.2408).

### Expression and purification of recombinant PvRBP1a

To express recombinant PvRBP1a proteins, the protocol followed that of a previous study [19]. In brief, overlapping fragments spanning the extracellular region of the RBP1a gene from the Sal1 allele (PVX_098585) were designed. The DNA sequences were codon optimised for *Escherichia coli* expression, commercially synthesized (Invitrogen, Carlsbad, CA, USA), and cloned into the pET21(a) +  expression vector with a C-terminal 6xHis-tag to facilitate purification. Protein expression was performed in *E. coli* BL21 (DE3) pLysE cells (Invitrogen). Bacterial cells were cultured in Luria–Bertani (LB) broth until reaching an OD_600_ of 0.6–0.8; protein expression was induced with 1 mM IPTG (final concentration) for 3 h at 30 °C. Cells were harvested by centrifugation at 11,000×*g* for 20 min and lysed in 20 mM phosphate buffer, pH 7.4, containing 0.5 M NaCl and 20 mM imidazole. Expressed proteins were purified by affinity chromatography, using Ni  +  Sepharose 6 fast flow (GE Life Sciences, Pittsburgh, PA, USA) according to the manufacturer’s recommendations. Purity of the antigens was checked by analysis via Coomassie-stained sodium dodecyl sulfate polyacrylamide gel electrophoresis (see Additional file 1) and then dialyzed against phosphate-buffered saline (PBS) before storage at − 80 °C.

### Total IgG and IgG subclass responses to PvRBP1a antigen

Anti-PvRBP1a antibody levels in plasma samples from patients during and after acute *P. vivax* infection were measured using an indirect ELISA. Briefly, 96-well microtiter plates were coated with 2 µg/mL recombinant PvRBP1a protein and incubated overnight at 4 °C. The plates were blocked with 5% skimmed milk in PBS-0.05% Tween for 2 h. The plasma samples at dilution 1:200 were then added in duplicate wells and incubated for 1 h. After washing, a 1:1000 dilution of goat anti-human IgG-alkaline phosphatase was added into the wells, and incubated for 1 h. Subsequently, ABTS substrate or 2,2-azino-bis (3-etylbenzthiazoline-6-sulfonic acid) (Sigma-Aldrich, St. Louis, MI, USA) was added to detect antigen–antibody reactivity. The Microplate Reader Synergy HTX (BIOTEK Instruments, USA) was used for reading absorbance at 405 nm. Two acute plasma samples with high titers were used as positive controls [19]. The OD values from duplicate wells per individual were averaged. A baseline OD was established using plasma samples from the 43 HCs.

To detect IgG subclass responses to RBP1a, a protocol similar to that for total IgG. In the step when each plasma sample was added at a dilution of 1:100, a modification used HRP-conjugated anti-human IgG1, IgG2, IgG3, and IgG4 antibodies at a dilution of 1:1000. Signal was developed with TMB (3, 3′5, 5′-tetramethylbenzidine) substrate (Merck KGaA, Sigma-Aldrich, USA). OD was read at 450 nm. Two seropositive samples for anti-PvRBP1a IgG response were used as positive controls. A baseline OD was established using plasma samples from 25 of the HCs.

The levels of total IgG or IgG subclasses specific to PvRBP1a were standardized to a reactivity index (RI), calculated by dividing OD values of tested samples by a cut-off value (mean  +  2SD) from HC samples. An RI  ≥  1 was considered to be positive for specific antibodies; those with an RI  <  1 were considered as non-responders (NRs). To distinguish levels of anti-PvRBP1a antibodies, a cut-off value was calculated as mean RI  +  0.5SD of all acute patients and used to classify high responder (HR; RI  >  mean  +  0.5SD) and low responder (LR; RI  <  mean  +  0.5SD but RI  ≥  1).

### Frequency of MBC subsets and plasmablasts/plasma cells

PBMCs collected during acute malaria were used for MBC subset and plasmablast/plasma cell phenotyping. Fluorochrome-conjugated, mouse anti-human monoclonal antibodies were used to stain 1 million PBMCs/100 μL FACS buffer. A cocktail consisting of the following mouse monoclonal antibodies was used: FITC-CD19, APC-CD21, APC/fire-CD27, AF700-CD38, PE-IgM and PE/Cy7-IgD (Biolegend, San Diego, CA, USA). After staining for 15 min, cells were washed with FACS buffer. Finally, cells were suspended in 250 μL FACS buffer. The analyses were done with a flow cytometer (FACSCanto II, Becton–Dickinson Immunocytometry Systems, San Jose, CA, USA). Data were processed using FlowJo software (Tree Star, San Carlos, CA, USA).

### ELISPOT assay

The presence of PvRBP1a-specific MBCs was determined by ELISPOT assay as performed in a previous study [25]. Briefly, 7 × 10^6^ PBMCs from *P. vivax* samples were prepared for cell stimulation along with negative controls. First, 1 × 10^6^ cells per mL were stimulated with a cocktail of polyclonal activator R848 (Invivogen) and recombinant human IL-2 (PrepoTech, NJ, USA) at 37 °C in a 5% CO_2_ incubator for 72 h. The ELISPOT assay was performed by coating with 15 μg/mL of monoclonal antibodies of anti-human IgG (clones MT91/145; Mabtech), 5 μg/mL of recombinant PvRBP1a antigen, or 1 μg/mL of tetanus toxoid (TT) antigen (Merck Millipore, Darmstadt, Germany) onto ELISPOT plates (Merck Millipore). Cells were harvested and seeded in duplicate to yield 5 × 10^4^ cells per anti-human IgG-coated well, and 1 × 10^6^ cells per specific antigen-coated well. After overnight culture, 1 μg/mL of detection monoclonal antibody MT78/145 (Mabtech) was added and incubated for 2 h. Following thorough washing, the diluted (1:1000) streptavidin-HRP-conjugated, polyclonal goat anti-human IgG (Mabtech) and TMB substrate for ELISPOT (Mabtech) were added. The plates were rinsed with deionized water after distinct spots emerged. PvRBP1a-specific ASCs were expressed as spot-forming cells (SFCs) in the wells. The plates were analysed with a CTL ELISPOT reader. PBMCs from *P. vivax*-infected subjects that were cultured without polystimulators and incubated overnight with antigens were used as negative controls. A positive PvRBP1a-specific MBCs response was defined as detectable spots in duplicate wells with the total spots in the specific antigen-coated wells being at least twice the number of spots detected with the negative control samples. The circulating PvRBP1a-specific ASCs were enumerated from PBMC cultures without polyclonal activation and the frequencies of ASCs were counted as spot forming units/1 million PBMCs. The antigen-uncoated wells were used as negative controls for circulating PvRBP1a-specific ASC analysis.

### Statistical analysis

The data were analysed using GraphPad Prism software (v. 8; GraphPad Software, San Diego, CA, USA). The Mann–Whitney U test was used to compare data from two groups of subjects (unpaired), while the Wilcoxon signed rank test was used to compare paired data from the same subjects at different time points. Kruskal–Wallis one-way ANOVA with Dunn’s multiple comparison was used to compare data from more than 2 groups. The relationship between the responses of PvRBP1a-specific MBCs by ELISPOT and antibodies by ELISA was done using a Fisher’s exact test. In all analyses, 2-tailed *P* values  < 0.05 were considered significant.

## Results

### Infections by *P. vivax* produced anti-PvRBP1a responses

**Fig. 1:**
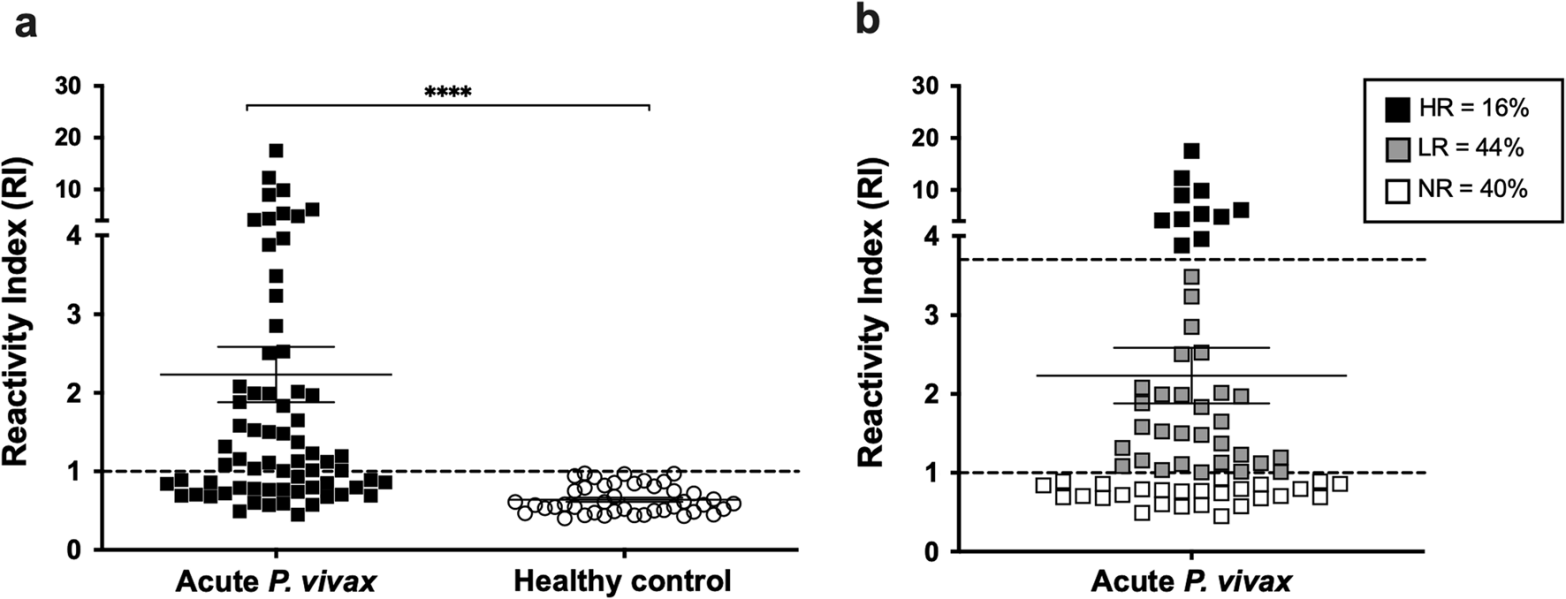
Antigenicity of PvRBP1a during acute *P. vivax* infection. **a** The reactivity index (RI) of total IgG antibody responses against PvRBP1a in acute *P. vivax* patients (n  = 68) and healthy controls (n  = 43) were detected using indirect ELISA. The horizontal line represents mean values  ±  SEM. Dash line indicates the cut-off value. ****P  < 0.0001. **b** The classification of subjects into 3 groups based on anti-PvRBP1a antibody levels, high responders (HR) indicate high antibody levels (n  = 11); low responders (LR) indicate low antibody levels (n  = 30); non-responders (NR) indicate seronegative response to PvRBP1a (n  = 27)

To determine the seroprevalence of anti-PvRBP1a antibody responses in natural infection to the *P. vivax* parasite, a cross-sectional survey of antibody levels against this antigen was conducted in acutely infected patients. During symptomatic malaria, anti-PvRBP1a IgG levels were significantly higher than in HCs (P  < 0.0001) (Fig. 1a). Positivity to PvRBP1a was 60.3% (41 of 68) in the group of acute patients (Fig. 1a). Based on the level of anti-PvRBP1a antibody during acute infections, the individuals were classified into 1 of 3 responder groups [HR (RI  > 3.7), LR (RI 1 to 3.7), and NR (RI  < 1)]. Among these subjects, 16, 44 and 40% were high, low and non-responders, respectively (Fig. 1b).

### IgG1 was the predominant subclass response to PvRBP1a

**Fig. 2:**
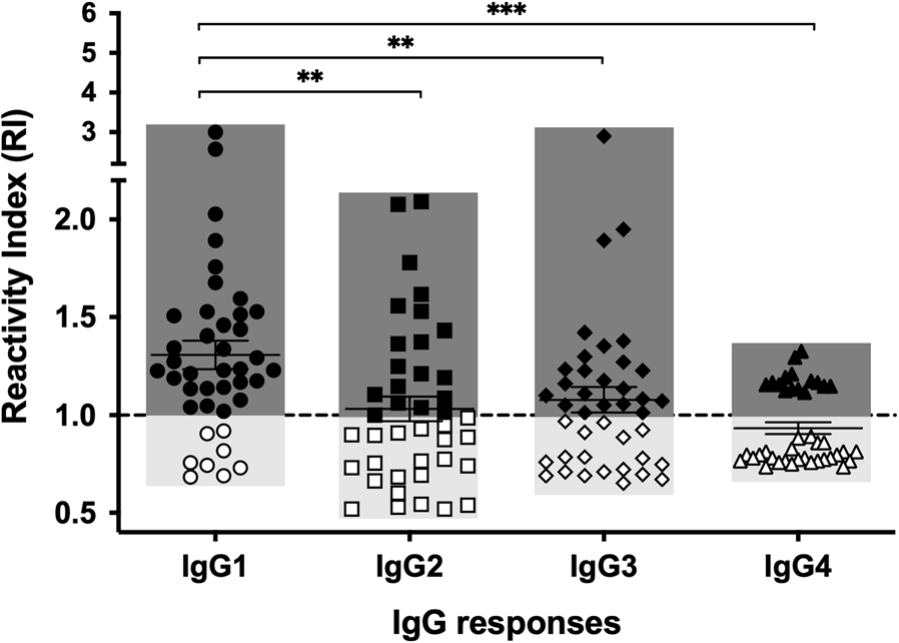
The seroprevalence of IgG subclass responses to PvRBP1a. The comparative anti-IgG1, -IgG2, -IgG3, and -IgG4 responses specific to PvRBP1a in seropositive subjects during acute malaria (n  = 41). The horizontal line represents mean values  ±  SEM. Dashed line indicates the cut-off value. Dark grey and light grey area represent seropositive and seronegative subjects, respectively. **P  < 0.01; ***P  < 0.001

Different IgG subclasses are involved in the protective humoral immunity to malaria [26, 27]. Thus, the relative anti-PvRBP1a IgG responses to each isotype in acute seropositive subjects were determined (Table 1). IgG1 was a significantly higher compared to IgG2, IgG3, IgG4 subclasses (Fig. 2). The seroprevalence of anti-PvRBP1a by IgG subclass was 82.93%, 48.78%, 58.54% and 41.46% for IgG1, IgG2, IgG3 and IgG4, respectively (Fig. 2). Thus, predominant subclass response to PvRBP1a antigen was IgG1.

### High responders maintained anti-IgG PvRBP1a seropositivity after infection

**Fig. 3:**
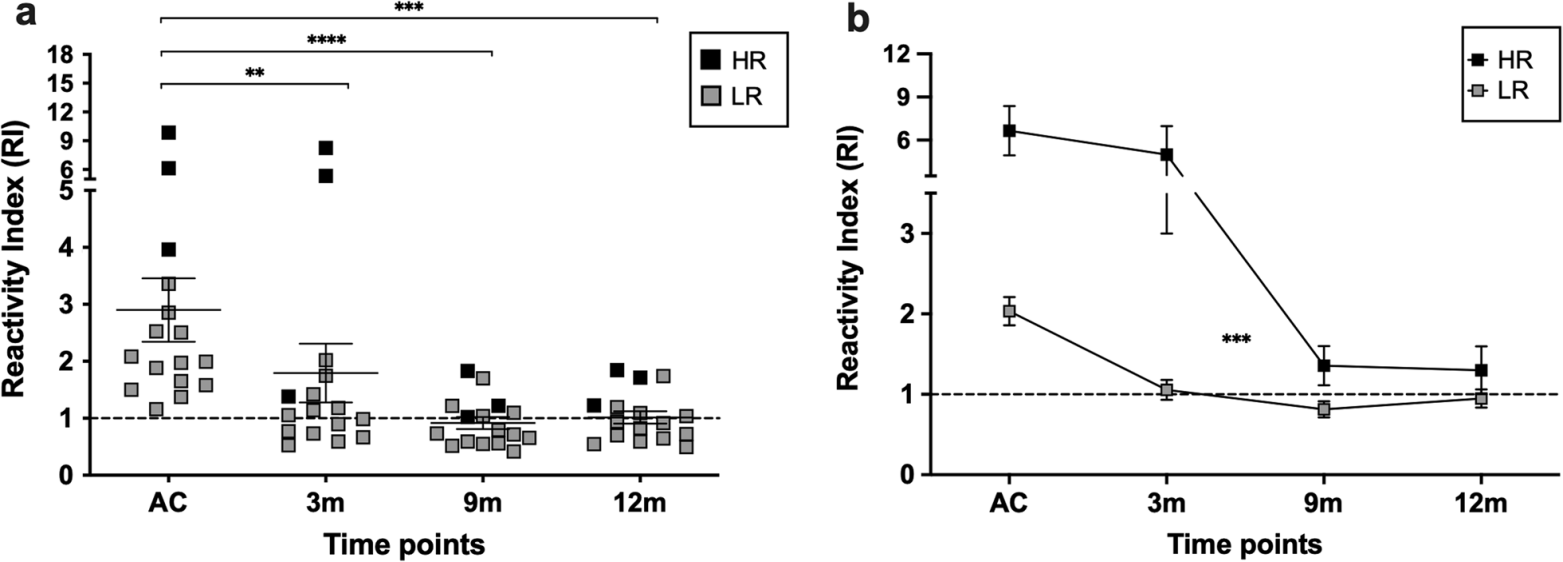
Longitudinal total IgG antibody responses against PvRBP1a. **a** The responses of anti-PvRBP1a antibody from 16 acutely *P. vivax*-infected subjects and at three follow-up times: 3 months (3m), 9 months (9m) and 12 months (12m) after infection. **b** Kinetic responses of the high responders (HR, n  = 3) and low responders (LR, n  = 13) at different time points (acute and recovered from infection for 3, 9 and 12 months). The horizontal line represents mean values  ±  SEM. **P  < 0.01; ***P  < 0.001; ****P  < 0.0001. *HR* high responders; *LR *low responders

To observe the kinetic of anti-PvRBP1a IgG responses after recovery from *P. vivax* infection, serial anti-PvRBP1a antibody levels were measured in 16 acute patients who were initially seropositive and agreed to additional blood collections (at 3, 9 and 12 months) (Table 1). At 3-month post-infection, total IgG anti-PvRBP1a levels were significantly reduced compared to the acute phase; responses continued to decline at the 9- and 12-month timepoints. There were 56.25% (9 of 16), 43.75% (7 of 16) and 37.5% (6 of 16) of the 16 subjects who maintained seropositive antibody responses to PvRBP1a at 3, 9 and 12 months, respectively (Fig. 3a). Further analysis of the response of these subjects in the kinetic study showed that most of the HR group (n  = 3, RI at acute  > 3.7) had persistent anti-PvRBP1a levels after parasite clearance through month 12, whereas antibody responses of the LR group (n  = 13, RI at acute 1–3.7) were significantly decreased at the 3-month timepoint (Fig. 3a, b).

### Expansions of activated and atypical MBCs were detected in high responders to PvRBP1a

**Fig. 4:**
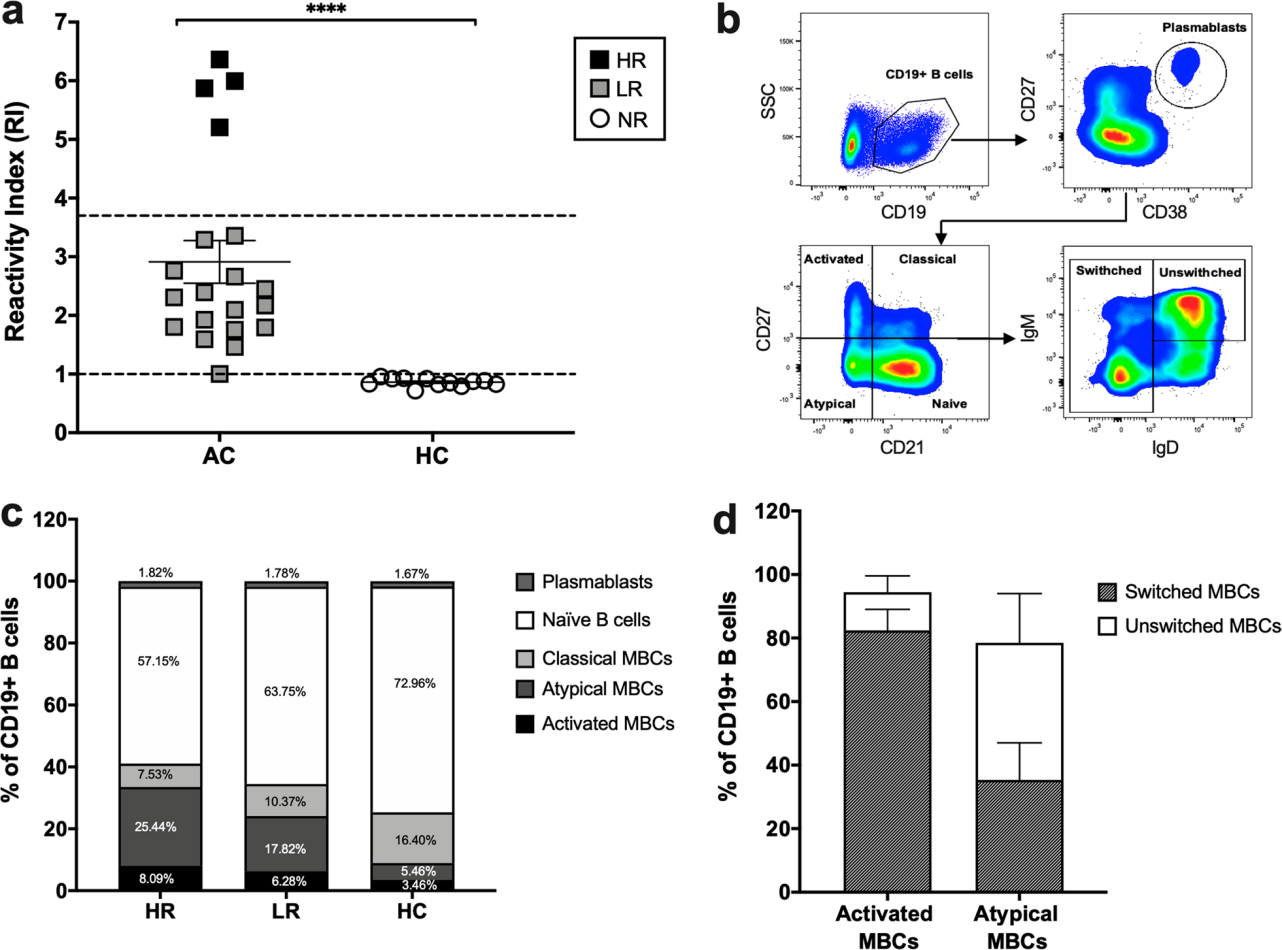
Responses of MBC subset and plasmablasts/plasma cells during acute malaria. **a** The antibody responses to PvRBP1a in acute patients (AC, n  = 20) and healthy controls (HC, n  = 12) were detected using an indirect ELISA. **b** Gating scheme for MBC subset and plasmablast/plasma cell phenotyping by flow cytometric analysis. Activated MBCs (CD19^+^CD21^−^CD27^+^), atypical MBCs (CD19^+^CD21^−^CD27^−^), classical MBCs (CD19^+^CD21^+^CD27^+^), naïve B cells (CD19^+^CD21^+^CD27^−^) and plasmablasts/plasma cells (CD19^+^CD27^hi^CD38^hi^). **c** The proportion of activated MBCs, atypical MBCs, classical MBCs, naïve B cells and plasmablasts/plasma cells detected in acute patients, high responders (HR, n  = 4) and low responders (LR, n  = 16) compared to healthy controls (HC, n  = 12). **d** The proportion of switched (IgM^−^IgD^−^ and IgM^+^ only) or unswitched (IgM^+^IgD^+^) MBC subpopulations were detected among activated and atypical MBCs in the HR group. The horizontal line represents mean values  ±  SEM. ****P  < 0.0001. *HR* high responders; *LR *low responders; *NR *non-responders

As HR patients showed the maintenance of anti-PvRBP1a antibody for at least 12 months, this could be due to the ability of PvRBP1a to induce development of MBCs during acute *P. vivax* malaria which persist after parasite clearance. To assess which MBC subsets might be related to anti-PvRBP1a antibody in *P. vivax* patients, an independent set of *P. vivax* subjects were studied, quantitating both anti-PvRBP1a IgG level and frequency of each MBC subset. Based on the levels of anti-PvRBP1a antibodies, 4 and 16 subjects were found to be HRs and LRs, respectively (Fig. 4a). To relate the antibody levels with MBC subset responses, the proportions of activated MBCs (CD21^−^CD27^+^), atypical MBCs (CD21^−^CD27^−^), classical MBCs (CD21^+^CD27^+^), naïve B cells (CD21^+^CD27^−^) and plasmablasts/plasma cells (CD27^hi^CD38^hi^) from total CD19^+^ B cells were determined (Fig. 4b) [28, 29]. Both HR and LR subjects showed an expansion of activated and atypical MBCs. In the HR group, 8.09% and 25.44% had activated and atypical MBCs, respectively. Whereas, lower proportions of activated (6.26%) and atypical MBCs (17.82%) were observed in the LR group (Fig. 4c). Moreover, the phenotyping showed that 82% of activated MBCs had the phenotype of switched MBCs (IgM^−^IgD^−^ and IgM^+^ only), whereas atypical MBCs showed the phenotypes of both switched and unswitched (IgM^+^IgD^+^) MBCs (35.33% of switched, 45.15% of un-switched MBCs) (Fig. 4d).

### Long-lived PvRBP1a-specific MBCs were detected up to 3-year post-infection

**Fig. 5:**
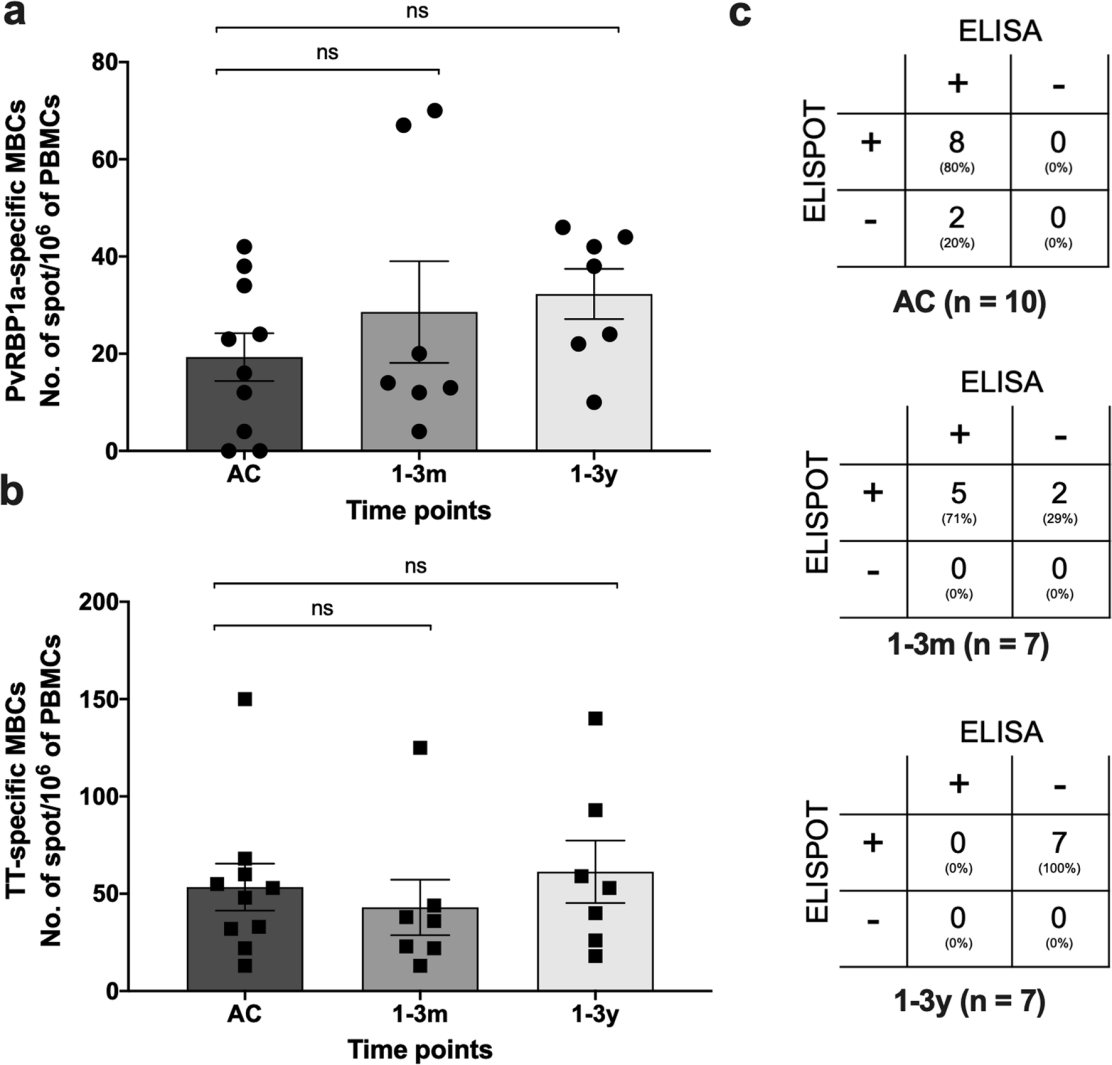
Development and persistence of PvRBP1a-specific MBCs. PvRBP1a‐specific MBCs detected during acute malaria and after recovery. **a** The number of specific MBC responses to the PvRBP1a antigens, **b** tetanus toxoid (TT) in PBMCs from patients during acute malaria (n  = 10) and after recovery (1–3 months, n  = 7; 1–3 years, n  = 7) were determined by ELISPOT (with polyclonal activation). The frequency of MBCs is expressed per million cultured PBMCs. Each symbol represents the MBC number for one individual. **c** Statistical analysis of association between the responses of PvRBP1a‐specific MBCs and antibodies in 10, 7 and 7 patients at acute and recovered phases for 1–3 months and 1–3 years, respectively. The horizontal line represents mean values  ±  SEM. Dashed line indicates the cut‐off value

To explore the presence of PvRBP1a-specific MBC responses, a cross-sectional study was conducted in acute (n  = 10) and recovered patients (1–3 months, n  = 7; 1–3 years, n  = 7). During acute malaria, 8 out of 10 patients had positive MBCs to PvRBP1a (SFCs ranging from 4 to 42). Post-infection, PvRBP1a-specific MBCs were detected in all subjects assessed 1–3-month post-infection (n  = 7; range, 4–70 SFCs) and 1–3-year post-infection (n  = 7; range, 10–46 SFCs) (Fig. 5a). As expected, nearly all subjects (95.83%) produced tetanus toxoid (TT)-specific MBCs (range, 13–150 SFCs) since TT commonly used as a routine vaccine and the high frequency of TT-specific MBCs was detected in vaccinated individuals (Fig. 5b). Analysis of the relationship between the responses of PvRBP1a-specific MBCs and antibodies within each patient showed that both individuals positive (80%) and negative (20%) for PvRBP1a-specific MBCs were seropositive for total IgG during acute infection (*P* value  = 1.00). For all positive specific MBCs at 1–3-month post-infection, 5 were seropositive and 2 were seronegative (*P* value  = 1.00). Interestingly, all seronegative subjects studied in 1–3-year post-infection had positive PvRBP1a-specific MBCs (*P* value  = 1.00) (Fig. 5c).

### Circulating PvRBP1a-specific ASCs were detected in subjects with acute *P. vivax* malaria

**Fig. 6:**
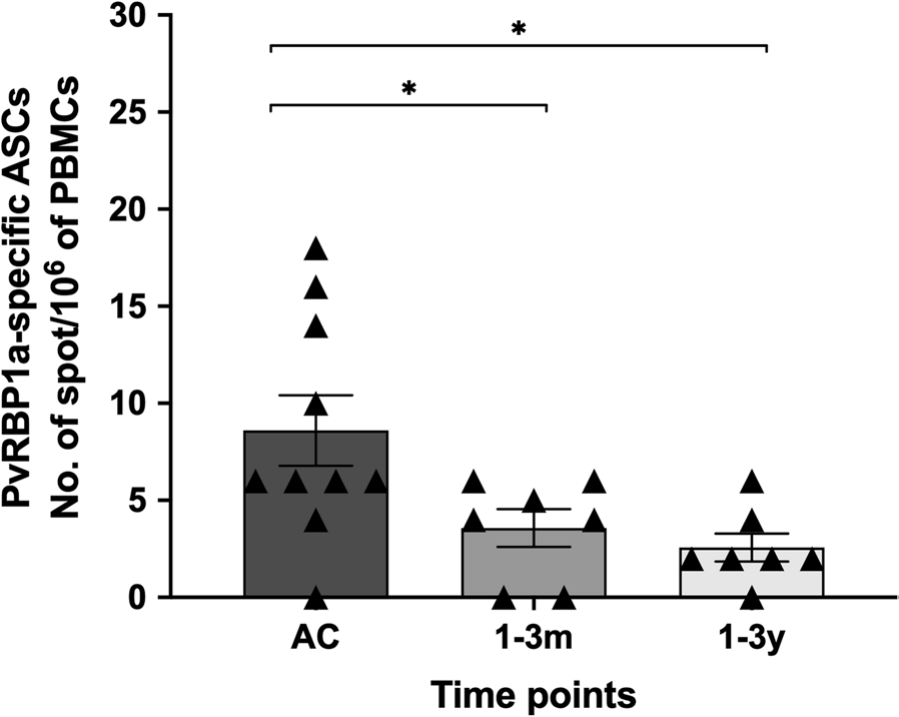
Circulating PvRBP1a-specific ASCs generated during acute infection. PMBCs from subjects during acute malaria (n  = 10) and recovered subjects (1–3 months, n  = 7; 1–3 years, n  = 7) were incubated directly ex vivo without polyclonal activation. The frequency of antigen-specific ASCs was expressed per million PBMCs. Each symbol represents the MBC number for one individual. The horizontal line represents mean values  ±  SEM. *P  < 0.05

To analyse the generation and persistence of ASCs specific to PvRBP1a after natural *P. vivax* infection, PBMCs from patients were cultured without polyclonal activation, incubated overnight with PvRBP1a and assessed by ELISPOT assay. During acute infection, 90% of patients (9 of 10) produced PvRBP1a-specific ASCs with SFCs ranging from 4 to 18. Following recovery, these subjects had few circulating ASCs specific to PvRBP1a (range, 0–6 SFCs) whether in the 1–3-month or 1–3-year post-infection window (Fig. 6).

## Discussion

PvRBP1a is one of the blood-stage antigens, expressed at the apical tip of merozoites which is shown to bind preferentially to reticulocytes and induces antibody responses [19, 21, 23, 30]. Here, the cross-sectional and longitudinal epidemiologic studies were designed to understand humoral immune response to PvRBP1a. The persistence of naturally acquired antibodies and MBC responses to PvRBP1a were observed in individuals who lived in an area of low malaria endemicity. Approximately 60.3% of *P. vivax*-infected patients were seropositive, predominately with the cytophilic IgG1 subclass, suggesting a high seroprevalence in such endemic areas. At post-infection, anti-PvRBP1a antibodies declined in detectability (56.25%, 43.75%, and 37.5% at 3-, 9- and 12-month post-infection, respectively). In contrast, MBC responses specific to the PvRBP1a antigen developed during acute disease and persisted for at least 3 years. These immunological characteristics suggest that PvRBP1a may be a good vaccine candidate, capable of inducing humoral responses against the blood stage of this malaria parasite.

Different IgG subclass responses have distinct immune effector functions, and these may be crucial for vaccine effectiveness [26, 27]. The cytophilic IgG1 and IgG3 subclasses are likely related to malaria protection by complement fixation and opsonization [31, 32]. The non-cytophilic IgG2 subclass correlates with control of parasitaemia by directly inhibiting parasite invasion and blocking cytoadherence of infected erythrocytes to endothelial cells [33, 34]. In this study, the results demonstrated that IgG1 was the predominant IgG subclass in the PvRBP1a responses to acute *P. vivax* infections. This is consistent with results that show predominantly cytophilic subclass IgG1 responses to PvRBP1a in individuals from Brazil and PNG [21, 24]. The IgG1 subclass antibodies to malaria antigens in naturally acquired immunity are associated with reduction in risk of malaria, with an apparent role in protective immunity [21, 35]. However, further studies are needed to evaluate functional inhibition of anti-PvRBP1a IgG1 against parasite invasion and the targeted epitopes of anti-PvRBP1a inhibitory antibodies, as well as to determine PvRBP1a-specific IgM response, alongside with IgG1. Since antibody responses often undergo class switching, and this may be affected by age, exposure to malaria, nature of the antigen and host factors, additional analyses will be needed to assess these immune processes.

The finding that a certain MBC subset plays a major role in producing long-term antibody responses is gold for developers of protective vaccines. Here, the HR subjects in the longitudinal study displayed long-lived responses of anti-PvRBP1a antibody, maintaining these responses for at least 12 months. This data suggested that PvRBP1a might induce the generation of MBCs during *P. vivax* infection which are maintained even after parasite clearance. Further analysis of association between MBC subsets and anti-PvRBP1a antibody responses in HR patients found higher proportions of activated and atypical MBCs but lower frequencies of classical MBCs during acute infection. This suggested that these activated and atypical MBCs expanded in response to the *P. vivax* infections. Similarly, expansions of activated and atypical MBCs occur in *P. falciparum* and *P. vivax* patients [28, 29, 36], as well as in HIV-infected individuals with high viral loads [37]. A possible explanation of these MBC subset expansions in HR patients is that classical MBCs were triggered via *P. vivax* infection and switched to activated MBCs to help in parasite clearance by antibody production [38, 39]. Alternatively, during activation of classical MBCs, atypical MBCs may arise in normal responses as a population of pre-ASCs originating from activation of naïve B cells [40, 41]. However, the data showed an expansion of the total population of activated and atypical MBC subsets without PvRBP1a specificity. Further studies are needed to evaluate in-depth the mechanisms by which MBC subsets are triggered by PvRBP1a, and lead to secretion of high anti-PvRBP1a antibody levels during infection. The use of PvRBP1a tetramer for analysis of phenotypic and functional features of PvRBP1a-specific MBCs as well as the expression levels of somatic hypermutation in PvRBP1a-specific switched IgG^+^ or unswitched IgM^+^ MBCs in primary and secondary responses is useful for vaccine strategies.

Long-lived MBCs and plasma cells provide a remarkably stable component of humoral immunity that can persist throughout life. Induction of both cell populations is important for improved vaccine strategies. In this study, most patients (80%) produced PvRBP1a-specific MBCs during acute infection, and these MBCs responses persisted in blood for at least 3 years after parasite clearance. However, the longevity of PvRBP1a-specific MBCs was not related to the duration of antibody responses. To get more understanding of the humoral immune response to PvRBP1a, ex vivo detection of circulating ASCs specific to PvRBP1a was performed during acute *P. vivax* malaria. Nearly all acute patients produced PvRBP1a-specific ASCs. After parasite clearance, significant decreases of circulating PvRBP1a-specific ASCs were observed. These findings suggest that PvRBP1a can induce the generation of MBCs and ASCs during *P. vivax* infection and that these cells are maintained in the absence of re-infection independent of the duration of antibody responses. A possible explanation could be that, upon infection with *P. vivax*, PvRBP1a antigen triggered naïve B cells. With helping signals from CD4^+^ T cells, activated B cells migrate to germinal centers where proliferation and selection take place. These processes could drive B cell differentiation into long-lived MBCs and ASCs [42, 43]. With subsequent parasite clearance, the PvRBP1a-specific MBCs remain in the blood circulation and so are able to respond to re-infections, while the specific ASCs might home to bone marrow [44, 45] or undergo apoptosis to negatively regulate their homeostasis [46]. Further studies are needed on a rare PvRBP1a-specific MBC subset and the plasma cell population in terms of their protective functions and longevity.

## Conclusions

In the present study, the ability of PvRBP1a in inducing the development and persistence of antibodies and MBCs was demonstrated in naturally *P. vivax*-infected individuals. The anti-PvRBP1a antibodies were maintained in HR patients for at least 12-month post-infection. Interestingly, circulating ASCs and MBCs specific to PvRBP1a were generated during *P. vivax* infection. The PvRBP1a-specific MBCs were stably detected up to 3 years after parasite clearance. Based on these findings, the PvRBP1a antigen should be developed as a potential candidate for a vivax malaria vaccine.

## Supplementary Information

**Additional file 1: Figure S1.** Production of recombinant PvRBP1a protein. Coomassie-stained sodium dodecyl sulfate polyacrylamide gel electrophoresis (SDS-PAGE) showed an elution fraction of rPvRBP1a. The recombinant PvRBP1a protein migrated as a single band at the expected mass of ~64 kDa on SDS-PAGE. Lane M is 10–250 kDa size marker; Lane E1–E7 are eluted protein of each fraction.

## Data Availability

The datasets used and/or analysed during the current study are available from corresponding author upon reasonable request.

## Abbreviations

PvRBP1a: *Plasmodium vivax* reticulocyte binding PROTEIN 1a
MBCs: Memory B cells
ASCs: Antibody-secreting cells
RBCs: Red blood cells
PBMCs: Peripheral blood mononuclear cells
