# Supplementary material for: The presence of circulating antibody secreting cells and long-lived memory B cell responses to reticulocyte binding protein 1a in Plasmodium vivax patients
Source: Malar J. 2021 Dec 20;20:474. doi: 10.1186/s12936-021-04015-3 (PMC8686587; doi:10.1186/s12936-021-04015-3)
Supplement: Supplementary file 1 — Additional file 1: Figure S1. Production of recombinant PvRBP1a protein. Coomassie-stained sodium dodecyl sulfate polyacrylamide gel electrophoresis (SDS-PAGE) showed an elution fraction of rPvRBP1a. The recombinant PvRBP1a protein migrated as a single band at the expected mass of ~64 kDa on SDS-PAGE. Lane M is 10–250 kDa size marker; Lane E1–E7 are eluted protein of each fraction. [file 12936_2021_4015_MOESM1_ESM.docx]

**The presence of circulating antibody secreting cells and long-lived memory B cell responses to reticulocyte binding protein 1a in *Plasmodium vivax* patients**

Piyawan Kochayoo^1^, Pattarawan Sanguansuttikul^1^, Pongsakorn Thawornpan^1^, Kittikorn Wangriatisak^1^, John H. Adams^2^, Francis B. Ntumngia^2*^ and Patchanee Chootong^1*^

^1^Department of Clinical Microbiology and Applied Technology, Faculty of Medical Technology, Mahidol University, Bangkok, 10700, Thailand.

^2^Center for Global Health and Infectious Diseases Research, University of South Florida, Tampa, Florida, 33612, USA.

*Corresponding authors

Patchanee Chootong, Ph.D., Email: [pchooton@gmail.com](mailto:pchooton@gmail.com); Tel: +66 2411 1096

Francis B. Ntumngia, Ph.D., Email: [fntumngi@usf.edu](mailto:fntumngi@usf.edu); Tel +813 974 0992

**Supplementary Information**

**
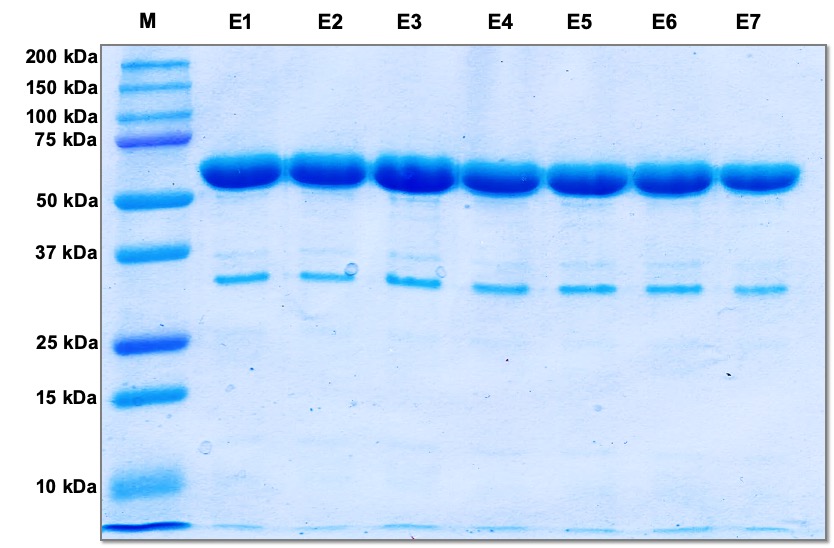
**

**Fig. S1.** Production of recombinant PvRBP1a protein. Coomassie-stained sodium dodecyl sulfate polyacrylamide gel electrophoresis (SDS-PAGE) showed an elution fraction of rPvRBP1a. The recombinant PvRBP1a protein migrated as a single band at the expected mass of ~64 kDa on SDS-PAGE. Lane M is 10-250 kDa size marker; Lane E1-E7 are eluted protein of each fraction.
